# An interview-based approach to assess sea turtle bycatch in Italian waters

**DOI:** 10.7717/peerj.3151

**Published:** 2017-04-26

**Authors:** Alessandro Lucchetti, Claudio Vasapollo, Massimo Virgili

**Affiliations:** Institute of Marine Sciences (ISMAR), National Research Council (CNR), Ancona, Italy

**Keywords:** Sea turtle bycatch, Interview-based approach, *Caretta caretta*, Sea turtle-fishery interaction, Zero-inflated analysis, Fishermen engagement, Mediterranean Sea

## Abstract

The loggerhead sea turtle (*Caretta caretta*, Linnaeus, 1758) is the most abundant sea turtle species in the Mediterranean Sea, where commercial fishing appears to be the main driver of mortality. So far, information on sea turtle bycatch in Italy is limited both in space and time due to logistical problems in data collected through onboard observations and on a limited number of vessels involved. In the present study, sea turtle bycatch in Italian waters was examined by collecting fishermen’s information on turtle bycatch through an interview-based approach. Their replies enabled the identification of bycatch hotspots in relation to area, season and to the main gear types. The most harmful fishing gears resulted to be trawl nets, showing the highest probabilities of turtle bycatch with a hotspot in the Adriatic Sea, followed by longlines in the Ionian Sea and in the Sicily Channel. Estimates obtained by the present results showed that more than 52,000 capture events and 10,000 deaths occurred in Italian waters in 2014, highlighting a more alarming scenario than earlier studies. The work shows that in case of poor data from other sources, direct questioning of fishermen and stakeholders could represent a useful and cost-effective approach capable of providing sufficient data to estimate annual bycatch rates and identify high-risk gear/location/season combinations.

## Introduction

Mediterranean fisheries are essentially multi-species and multi-gear. Fishing fleets consist mostly of medium to large, highly differentiated, competing vessels that often exploit shared resources (Lucchetti et al., 2014). Intense and prolonged fishing pressure has resulted in overexploitation of fish resources (Colloca et al., 2013) and deterioration of marine ecosystems (Tudela, 2004; Sacchi, 2008). Large vertebrates like sharks (Ferretti et al., 2008), cetaceans (Bearzi, 2002), monk seals (Karamanlidis et al., 2008) and, above all, sea turtles (Casale, 2011) are the most affected species. These species are particularly vulnerable for biological reasons including late maturity and low reproduction rates.

The loggerhead sea turtle (*Caretta caretta*, Linnaeus, 1758) is the most abundant sea turtle species in the Mediterranean Sea (Casale & Margaritoulis, 2010 and references therein; Lucchetti & Sala, 2010). However, it is a priority species in Appendix II/IV of the Habitats Directive, the cornerstone of the EU nature conservation policy, which lists animals requiring close protection (EU, 1992). *C. caretta* is also included in the red list of the International Union for the Conservation of Nature and Natural Resources (Casale & Tucker, 2015). Although recent assessments have downgraded this species from the *status* “endangered” to “vulnerable” at a global scale, the adoption of conservation actions was stressed as crucial point. Its conservation has become a strategic issue in the whole Mediterranean, where commercial fishing appears to be the main driver of mortality for marine turtles (Lucchetti & Sala, 2010; Casale, 2011; Wallace et al., 2011).

Due to their habits (e.g., breeding and feeding migrations), loggerhead turtles interact with several types of fishing gears (e.g., demersal and pelagic towed gears, set nets, longlines; Wallace et al., 2008; Lucchetti & Sala, 2010). In the Mediterranean turtle bycatch is mainly related to the three main fishing methods adopted in the region (Lucchetti & Sala, 2010; Casale, 2011): drifting longlines (Guglielmi, Di Natale & Pelusi, 2000; Piovano et al., 2004; Deflorio et al., 2005; Jribi et al., 2008; Tomás et al., 2008; Piovano, Swimmer & Giacoma, 2009; Clusa et al., 2016), trawling (Casale, Laurent & De Metrio, 2004; Jribi, Bradai & Bouain, 2007; Sala, Lucchetti & Affronte, 2011; Domènech et al., 2015; Lucchetti et al., 2016), and set nets (Lazar, Ziza & Tvrtkovic, 2006; Echwikhi et al., 2010).

Longline bycatch occurs in open waters during the pelagic stage of the loggerhead turtle life, with high rate areas in Spanish (Báez et al., 2007; Clusa et al., 2016), North African (Jribi et al., 2008; Benhardouze, Aksissou & Tiwari, 2012), Greek (Snape et al., 2013), and southern Italian waters (Piovano et al., 2012). Bycatch events involve attraction by bait, hooking, and attempts to escape. Delayed mortality due to lesions caused by the swallowing of hooks and branch lines is a major concern and is suspected to be high (Casale, Freggi & Rocco, 2008).

Bottom trawling mostly interferes with the demersal stage. In the Mediterranean Sea, the main neritic habitats are found in the few, large, continental shelf areas, i.e., the Northern Adriatic Sea, the Gulf of Gabès, Egypt, and East Turkey (Lucchetti & Sala, 2010), where turtles in the demersal stage are more likely to assemble in shallow water, in order to feed on the abundant prey near the bottom. According to several studies (Henwood & Stuntz, 1987; Sasso & Epperly, 2006), direct mortality due to trawling depends on tow duration and hence to the submergence time, being high with prolonged apnoea. However, delayed mortality due to drowning, metabolic disturbance, decompression sickness upon release (García-Párraga et al., 2014) and the possibility of re-capture is suspected to be high.

Set nets are a risk for turtles in the neritic stage. Interactions mainly take place in coastal areas, where they seem to be considerable and comparable to those occurring in other fisheries. However, considering the wide diffusion of set nets, interactions may actually be greater. Data are scarce. Mortality induced in this case is related to forced apnoea and consequent drowning due to the high soak time of the nets. The available data suggest that the mortality observed at the time of gear retrieval is very high (Lucchetti & Sala, 2010).

Over the past 10 years, satellite tracking has provided important information on many aspects of the biology, ethology, distribution and migration routes of *C. caretta* (Hays et al., 1991; Hays, 1992; Godley et al., 2003; Zbinden et al., 2008; Casale et al., 2012; Luschi & Casale, 2014; Lucchetti et al., 2016) and bycatch estimates in the Mediterranean Sea have been reported for several countries and fishing gears. Seasonal variation in turtle density and abundance can also be studied using aerial surveys; however, these techniques are expensive and can be strongly affected by the presence of turtles on the sea surface during the surveys (Lauriano et al., 2011; Bovery & Wyneken, 2015). In Italy bycatch assessment often suffers of logistical problems in data collection, since the information is usually obtained from on board observations involving short periods of time, a limited number of vessels, and small area covered; moreover, sampling procedures are often not standardized (Dmitrieva et al., 2013). The approach is time-consuming and cost-intensive, and reliable information can only be obtained by fielding a massive sampling effort. Moreover, data from some fisheries are particularly difficult to obtain due to difficult observer access or inadequate monitoring. As a result, reviews of sea turtle bycatch in the Mediterranean (Lucchetti & Sala, 2010; Casale, 2011) are largely based on onboard observations and only rarely on logbooks.

An alternative approach to estimate turtle bycatch by direct interviews of fishermen has been adopted in Spain giving promising results regarding the reliability of the derived bycatch rate estimates (De Quevedo et al., 2010; Domènech et al., 2015). Over the past decade, social studies have explored the fishermen’s perspective in view of the design of innovative management approaches (Griffin, 2009; Lucchetti et al., 2014; Santiago et al., 2015). More recently, the bottom up-approach (fishermen’s perspective and stakeholder engagement) has also been applied in biological and ecological studies (Lewison et al., 2011; Kiszka, 2012; Nguyen et al., 2013) to examine several issues, including fisheries bycatch.

The aim of this study is twofold: (1) to estimate sea turtle bycatch rates in the Italian waters adopting an interview-based approach, with the involvement of fishermen; (2) to assess the implicit risk of bycatch for each kind of fishing gear, identifying possible seasonal and spatial bycatch hotspots.

### Material and Methods

### Study area

**Figure 1:**
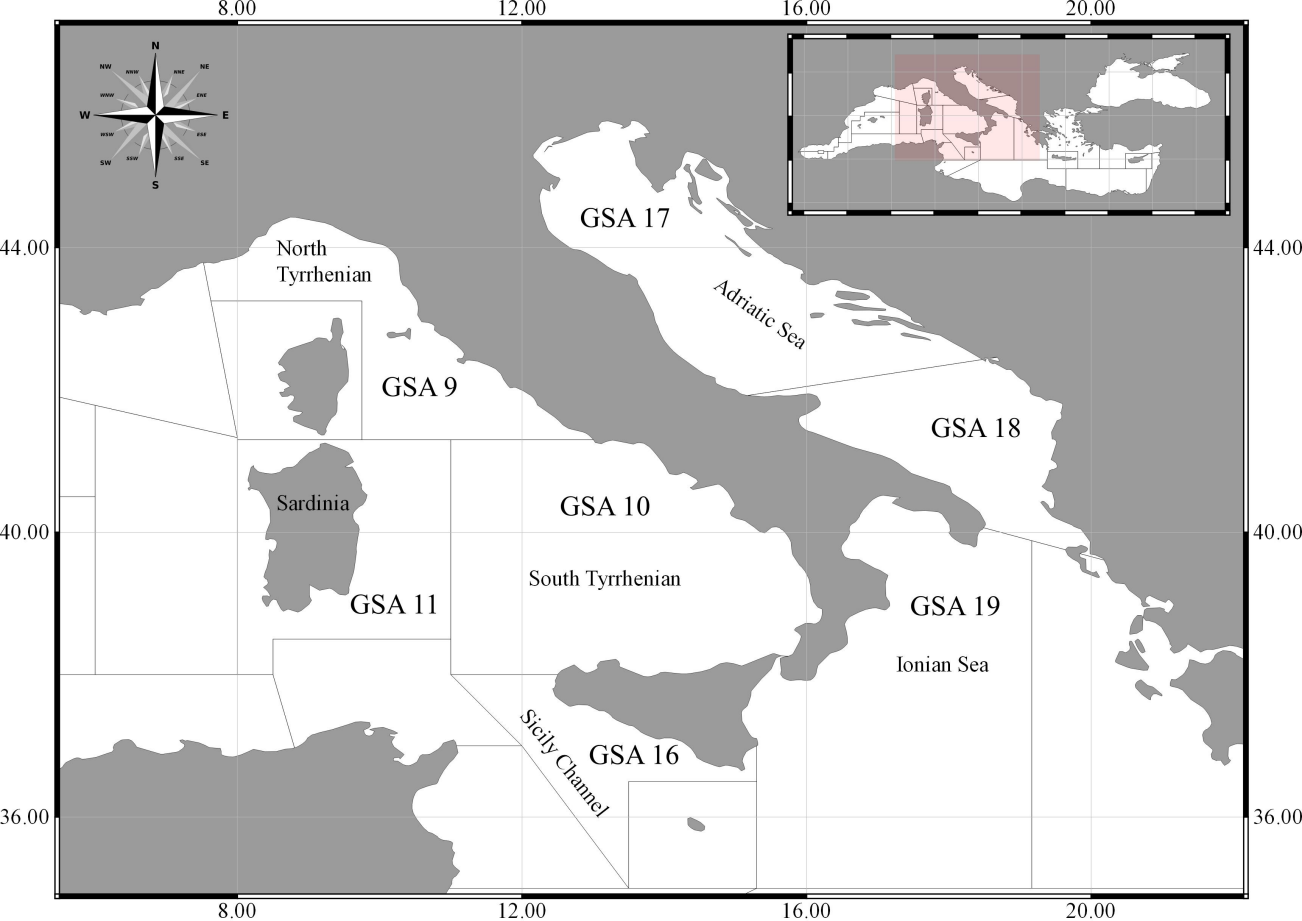
Study area. Mediterranean GSAs (Geographical Sub-Areas) involved in the data collection and questionnaires.

The study was carried out in Italy (central Mediterranean Sea, Fig. 1), which encompasses loggerhead migration routes (Sicily channel and Ionian Sea; Bentivegna, 2002), foraging areas (Adriatic Sea; Casale et al., 2012) and stable nesting rookery in the Ionian Sea (Mingozzi et al., 2007; Garofalo et al., 2009). Data are presented by Geographical Sub-Areas (GSAs) of the General Fisheries Commission for the Mediterranean comprising the Italian coastline, assuming that the bycatch relating to each fishing method and environmental condition in each GSA are sufficiently similar to provide a homogenous bycatch amount. The data from each GSA were then divided by fishing method and season.

### Interview survey design

More than 30 interviewers from 9 institutions from different parts of Italy, including a research body, a regional authority, 2 non-profit organizations, 2 private organizations, 2 marine protected areas and 1 national park, participated in the survey. In total 453 interviews were conducted in 105 Italian fishing harbours, covering all Italian GSAs and about 98% of the Italian coast length (total 7,458 km). About 6% of the entire Italian fleet was interviewed. Sampling distribution across geographical areas was generally in line with the distribution of the fishing fleet, even though the consistency of data collection and reporting varied across GSAs and fishing methods.

Face to face interviews were organized directly in the harbour, on board fishing vessels and during fishermen’s associations meetings. A single questionnaire was used for all fisheries and fishing methods.

The interview consisted of 5 sections: (1) ‘*background information’* involved questions related to the fisherman’s experience, fishing gear used and fishing grounds; (2) ‘*frequency of turtle encounters and fishermen’s behaviour*’ involved questions on the number of sea turtles caught per season in 2014 and on their management upon capture; (3) ‘*suggestions to reduce turtle bycatch and knowledge of bycatch reducer devices*’ asked how the interviewee thought that turtle bycatch could be reduced and for his opinion on the adoption of Bycatch Reducer Devices (BRDs) and gear modifications (such as circle hooks for longlines, Turtle Excluder Devices for trawl nets or light deterrent for passive nets); (4) ‘*fishermen’s awareness and attitude regarding turtle conservation*’, asked about the interviewee’s willingness to pursue responsible fishing and turtle conservation; and, finally, (5) ‘*participation and cooperation*’, tested their real interest in participating and cooperating in turtle conservation initiatives and research projects.

The questionnaire was designed to be completed in 15 min. Most questions were closed, which allowed collecting quantitative and factual information; some questions exploring fishermen’s opinion, in sections (3) and (5), were multiple choice questions that allowed greater freedom to their answers.

### Ethics statement

All necessary permits were obtained for the field studies described. Interviewees were informed of the purpose of the study and that the data collected were confidential, and that their anonymity would be protected. The interviews were carried out only after fishermen verbally consented to participate.

### Sampling methods

The study included the fishing gear types most commonly used, which were identified by a literature search (Lucchetti & Sala, 2010; Casale, 2011); those for which bycatch had been reported sporadically or not at all (i.e., purse seines) were excluded. Then, the fishermen to be interviewed were identified via opportunistic and ‘snowball’ sampling, which is widely used in sociological research (Biernacki & Waldorf, 1981; Faugier & Sargeant, 1997) and consists in the recruitment of future subjects from the acquaintances of fishermen interviewed before, overtaking the initial typical fisherman’s confidentiality. This kind of sampling permitted to reach a reasonable number of interviews. Fishermen were approached at local harbours or in public places. Since crews generally consist of several fishermen, one single fisherman per vessel was interviewed.

### Data analysis

The data collected in the study related to 2014. Two types of information are required to obtain quantitative estimates and spatial data on bycatch (Moore et al., 2010): the measure of the fishing effort and the bycatch rate.

Data on the fishing effort were obtained from the EU Data Collection Framework (EU, 2008a), set up in 2000, through which Member States collect, manage, and make available a wide range of fisheries data (including biological and socio-economic data) that are needed to obtain scientific advice. Data were collected on the basis of national programmes. Disaggregated data from the DCF dataset, provided by the Italian Ministry for Agricultural, Food and Forestry Policies, were extrapolated to obtain two indexes of fishing effort: the *total number of fishing vessels* operating in each season in the Italian GSAs and the *number of days at sea* recorded in 2014.

Data on sea turtle bycatch were obtained from interviews with fishermen. The bycatch estimates, per GSA and season, were obtained by averaging (geometric mean) the number of turtles reported by fishermen for each GSA, season and gear. The geometric mean was considered to smooth the effect of the extreme values. Then, the *average number of turtles* caught per season, area and gear from a single boat was multiplied by the *total number of vessels* that actually worked with that gear in each season and area, to obtain the total number of turtles caught in Italian waters.

Data from interviews were considered as valid only if, for each combination of gear-season-GSA, at least 5 interviews were performed. Turtle bycatch was rated on a scale from 1 (low bycatch: 0–100 turtles) to 6 (very high bycatch > 1,000 turtles).

Fishermen were also asked to report the percentage of death turtles at the end of the gear retrieval (mortality rate). This value was considered to estimate the number of deaths (*estimated turtle bycatch × mortality rate*). The mortality rate and the estimated death of turtles obtained in the current study were then compared with those reported by Casale (2011), who made a complete review of the mortality rates for different areas of the Mediterranean Sea.

Due to the nature of data obtained by the interviews, characterized by an excess of zeros (about 75% of the entire dataset), a zero inflated model regression analysis based on negative binomial distribution (ZINB) with logit link was performed to reduce overdispersion of variance due to the zeros (Zuur et al., 2009). In ZINB analysis the zeros and the counts are analysed as two different datasets: a binomial generalized linear model (GLM) is used to model the probability of measuring a zero (called false zero, generally due to design errors, observer errors, unsuitable habitat and so on); the count process was modelled by a negative binomial GLM and as such, under certain covariate conditions, can produce zeros (in the sense that we can count zero turtles effectively and referred as true, or structural zeros). The expected mean and variance for the ZINB model are calculated as follow:

\begin{eqnarray*}E \left( {Y}_{i} \right) ={\mu }_{i}\times \left( 1-{\pi }_{i} \right) \end{eqnarray*}

\begin{eqnarray*}var \left( {Y}_{i} \right) = \left( 1-{\pi }_{i} \right) \times \left( {\mu }_{i}+ \frac{{\mu }_{i}^{2}}{k} \right) +{\mu }_{i}^{2}\times \left( {\pi }_{i}^{2}+{\pi }_{i} \right) \end{eqnarray*}

where $E \left( {Y}_{i} \right) $ is the expected value of the response variable, *μ*_*i*_ is the mean of the positive count data and *π*_*i*_ is the probability to have false zeros. We also calculated the probability functions of ZINB to have zeros (true zeros) and negative binomial distribution for the count data as follows:

\begin{eqnarray*}f \left( {y}_{i}=0 \right) ={\pi }_{i}+ \left( 1-{\pi }_{i} \right) \times { \left( \frac{k}{{\mu }_{i}+k} \right) }^{k} \end{eqnarray*}

\begin{eqnarray*}f \left( {y}_{i}{|}{y}_{i}\gt 0 \right) = \left( 1-{\pi }_{i} \right) \times \frac{ \left( {y}_{i}+k \right) {!}}{ \left( k \right) {!}+ \left( {y}_{i}+1 \right) {!}} \times { \left( \frac{k}{{\mu }_{i}+k} \right) }^{k}\times { \left( 1- \frac{k}{{\mu }_{i}+k} \right) }^{y} \end{eqnarray*}

where *k* is called the dispersion parameter.

The covariates considered for modelling data were GSA (7 levels), Season (4 levels) and Gear (3 levels). The model selection was performed following a backward selection of covariates starting from a full model with all the covariates and interactions and assessed via Akaike’s Information Criterion (AIC); the model with lowest AIC was considered the best. To assess the significance of each single factor and interactions a likelihood ratio test (LRT) based on Chi^2^ distribution was used dropping each term in turn (Zuur et al., 2009). The best model selected consisted in the interaction between Gear and GSA (GSA × Gear) in the “count” part and the interaction between GSA and Season plus Gear as single factor (GSA × season + gear) in the “zero” part. The statistical analysis were conducted with R (v. 3.3.2; R Core Team, 2016) using the *pscl* package (v. 1.4.9; Zeileis, Kleiber & Jackman, 2008).

Data on stranded turtles, obtained from the Coast Guard Marine Environment Department (Reparto Ambientale Marino) database of the Italian Ministry of the Environment, were also considered as a rough index of turtle presence and abundance, and were ranked using a 6-point scale from 1 (low strandings: 0–25 turtles) to 6 (high strandings >150 turtles), to confirm the presence of hotspot areas and periods.

An interaction matrix was finally developed to find hotspot areas and periods of interaction between fishing gears and turtles for each gear. For the calculation of this matrix, fishing effort was expressed as *total number of fishing days per season*. The matrix was then calculated by dividing the average catch obtained by GSA, gear and season for the fishing effort expressed as *total number of fishing days*. The bycatch-effort interaction was also ranked from 1 (lowest risk of interaction, 0.000–0.018) to 6 (highest risk of interaction, >0.08).

The data on fishing effort, bycatch, stranded turtles and interaction matrix were plotted on separate maps using QGIS 2.8 software.

## Results

### Sea turtle bycatch estimates

The fishing effort per gear type (fishing days/season/gear) calculated in the different GSAs varied greatly among seasons and gears (Table 1). For set nets it seemed to be higher in GSA 19 (Ionian Sea), given the large number of vessels operating there, especially in spring and summer. For trawling, the fishing effort was the highest in GSA 17 (Northern Adriatic Sea), where the low depth, flat seabed is ideal for towed gears, but fell in summer due to the closed fishing season. The fishing effort of longlines was consistently low, except in GSA 19 in summer.

**Table 1:** Fishing effort. Indexes of fishing effort calculated in 2014: Number of vessels (NV) and Fishing days per season (FD) in each GSA divided by fishing gear (Longlines, Set nets, Trawls).

Gear	GSA	Spring		Summer		Autumn		Winter		Total	
		NV	FD	NV	FD	NV	FD	NV	FD	NV	FD
Longlines	GSA 09 - NT	65	2,131	122	3730	50	623	33	931	270	7,416
	GSA 10 - ST	150	7,034	181	5,896	50	1,157	31	388	412	14,475
	GSA 11- SR	25	818	49	1,503	16	82	18	359	108	2,762
	GSA 16 - SC	54	1974	64	2,605	31	201	27	321	176	5,101
	GSA 17 - NA	15	479	13	355	15	153	16	328	59	1,315
	GSA 18 - SA	8	426	8	574	8	113	8	70	32	1,182
	GSA 19 - IS	86	5,432	165	14,480	146	8,656	54	2,209	451	30,777
	Total	403	18,295	602	29,142	316	10,984	187	4,606	1,508	63,026
Set nets	GSA 09 - NT	1,024	36,148	950	31,674	799	22,382	870	26,408	3,643	116,612
	GSA 10 - ST	1,341	46,388	1,168	46,126	1,007	32,798	1143	31,155	4,659	156,467
	GSA 11- SR	1,096	33,978	977	31,981	597	11,968	687	16,162	3,357	94,088
	GSA 16 - SC	475	17,237	502	17,104	276	5,134	293	7,849	1,546	47,323
	GSA 17 - NA	862	22,668	1,048	32,265	821	20,306	697	15,968	3,428	91,208
	GSA 18 - SA	417	17,140	348	17,984	298	11,647	359	8,798	1,422	55,569
	GSA 19 - IS	1,095	49,586	1,153	58,907	1,020	38,976	1,144	43,137	4,412	190,605
	Total	6,310	223,145	6,146	236,040	4,818	143,211	5,193	149,477	22,467	751,873
Trawl nets	GSA 09 - NT	208	14,476	206	13,144	181	10,495	193	12,874	788	50,988
	GSA 10 - ST	280	12,763	260	11,084	270	9,504	296	11,221	1,106	44,572
	GSA 11- SR	97	4,043	102	3,157	101	2,412	100	3,862	400	13,475
	GSA 16 - SC	288	18,066	305	15,424	233	10,338	294	14,174	1,120	58,002
	GSA 17 - NA	596	26,915	471	16,053	539	23,844	605	27,194	2,211	94,006
	GSA 18 - SA	443	14,671	429	10,299	428	14,501	427	13,227	1,727	52,697
	GSA 19 - IS	248	10,885	241	9,015	264	7,646	270	9,025	1,023	36,571
	Total	2,160	101,819	2,014	78,176	2,016	78,739	2,185	91,577	8,375	350,311

**Notes.**

NT North Tyrrhenian ST South Tyrrhenian SR Sardinia SC Sicily Channel NA North Adriatic SA South Adriatic IS Ionian Sea

The mean number (geometric mean) of turtles captured per GSA, fishing gear and season, obtained by the fishermen interviewed, is reported in Table 2.

**Table 2:** Mean turtle bycatch. Mean turtle bycatch per vessel (geometric mean and standard error: se) obtained from the interviews per GSA, fishing gear and season.

		Spring		Summer		Autumn		Winter	
		Mean	se	Mean	se	Mean	se	Mean	se
Longlines	GSA 09 - NT	0.0	0.0	7.4	9.5	24.5	6.3	1.0	0.2
	GSA 10 - ST	1.2	0.2	0.0	0.0	2.0	0.2	0.0	0.0
	GSA 11 - SR	0.0	0.0	1.5	0.3	0.0	0.0	0.0	0.0
	GSA 16 - SC	2.0	0.3	15.0	2.5	3.9	2.5	0.0	0.0
	GSA 17 - NA	0.0	0.0	14.0	2.1	0.0	0.0	0.0	0.0
	GSA 18 - SA	1.0	0.0	11.0	1.8	0.0	0.0	0.0	0.0
	GSA 19 - IS	6.3	1.7	5.5	5.4	17.3	3.5	9.3	1.8
Set nets	GSA 09 - NT	1.4	0.2	1.3	0.3	1.0	0.1	1.0	0.1
	GSA 10 - ST	1.6	0.2	1.7	0.1	1.1	0.1	1.8	0.1
	GSA 11 - SR	0.0	0.0	1.4	0.2	0.0	0.0	0.0	0.0
	GSA 16 - SC	1.0	0.1	0.0	0.0	0.0	0.0	0.0	0.0
	GSA 17 - NA	2.0	0.2	1.8	0.2	2.0	0.1	1.6	0.0
	GSA 18 - SA	1.0	0.3	1.6	0.5	5.0	1.3	1.0	0.3
	GSA 19 - IS	1.0	0.1	0.0	0.0	0.0	0.0	0.0	0.0
Trawl nets	GSA 09 - NT	1.4	0.2	1.3	0.2	1.3	0.2	1.0	0.1
	GSA 10 - ST	0.0	0.0	0.0	0.0	0.0	0.0	2.0	0.0
	GSA 11 - SR	0.0	0.0	1.0	0.1	1.0	0.1	0.0	0.0
	GSA 16 - SC	2.0	0.7	3.0	1.0	2.0	0.7	3.0	1.0
	GSA 17 - NA	3.8	0.4	3.4	0.4	4.1	0.4	4.2	0.4
	GSA 18 - SA	3.4	0.5	4.4	0.5	3.8	0.8	2.5	0.9
	GSA 19 - IS	0.0	0.0	1.0	0.2	0.0	0.0	2.0	0.4

**Notes.**

NT North Tyrrhenian ST South Tyrrhenian SR Sardinia SC Sicily Channel NA North Adriatic SA South Adriatic IS Ionian Sea

Relying on interview data 52,340 capture events are estimated to occur in 2014 (Table 3). The majority of incidental catches took place in summer (>15,000 events), followed by autumn and spring (around 13,600 and 13,000 respectively), whereas a lower number were caught in winter (around 11,000) (Table 2). Catches by trawl nets mainly occurred in GSAs 17 and 18 (Adriatic Sea), where they seemed to be numerous throughout the year. Longline bycatch mainly occurred in GSAs 19 and 16 (Southern Italy), especially in summer and, to a lesser extent, in autumn. Set nets seemed to interact with turtles in most GSAs especially in spring and summer, when fishing with this gear is most active due to favourable sea and weather conditions.

**Table 3:** Estimate of capture events. Estimate of capture events and turtle deaths per GSA, fishing gear and season. The mortality rate obtained from interviews (current paper) and from Casale (2011) were used to calculate the estimates of turtle deaths.

		Spring			Summer			Autumn			Winter			Total		
		Bycatch	Mortality		Bycatch	Mortality		Bycatch	Mortality		Bycatch	Mortality		Bycatch	Mortality	
			Current	Cas. 2011		Current	Cas. 2011		Current	Cas. 2011		Current	Cas. 2011		Current	Cas. 2011
Longlines	GSA 09 - NT	0.0	0.0	0.0	902.9	125.3	270.9	1224.7	169.9	367.4	33.0	4.6	9.9	2160.6	299.8	648.2
	GSA 10 - ST	175.0	24.3	52.5	0.0	0.0	0.0	100.0	13.9	30.0	0.0	0.0	0.0	275.0	38.2	82.5
	GSA 11 - SR	0.0	0.0	0.0	71.2	9.9	21.4	0.0	0.0	0.0	0.0	0.0	0.0	71.2	9.9	21.4
	GSA 16 - SC	108.0	15.0	32.4	960.0	133.2	288.0	120.1	16.7	36.0	0.0	0.0	0.0	1188.1	164.8	356.4
	GSA 17 - NA	0.0	0.0	0.0	182.0	25.3	54.6	0.0	0.0	0.0	0.0	0.0	0.0	130.0	25.3	54.6
	GSA 18 - SA	8.0	1.1	2.4	88.0	12.2	26.4	0.0	0.0	0.0	0.0	0.0	0.0	96.0	13.3	28.8
	GSA 19 - IS	543.9	75.5	163.2	914.0	126.8	274.2	2532.4	351.4	759.7	501.3	69.6	150.4	4491.5	623.2	1347.5
	**Total**	**834.9**	**115.8**	**250.5**	**3118.1**	**432.6**	**935.4**	**3977.2**	**551.8**	**1193.2**	**534.3**	**74.1**	**160.3**	**8412.4**	**1174.4**	**2539.3**
**Set nets**	GSA 09 - NT	1460.4	34.3	438.1	1196.9	28.1	359.1	799.0	192.6	479.4	870.0	209.7	522.0	4326.3	1042.6	2595.8
	GSA 10 - ST	2194.0	51.6	658.2	2033.6	47.8	610.1	1156.7	278.8	694.0	2077.0	500.5	1246.2	7461.3	1798.2	4476.8
	GSA 11 - SR	0.0	0.0	0.0	1365.0	32.1	409.5	0.0	0.0	0.0	0.0	0.0	0.0	1365.0	329.0	819.0
	GSA 16 - SC	475.0	11.2	142.5	0.0	0.0	0.0	0.0	0.0	0.0	0.0	0.0	0.0	475.0	114.5	285.0
	GSA 17 - NA	1731.1	40.7	519.3	1840.1	43.2	552.0	1628.8	392.5	977.3	1090.9	262.9	654.5	6290.9	1516.1	3774.5
	GSA 18 - SA	417.0	9.8	125.1	552.4	13.0	165.7	1490.0	359.1	894.0	359.0	86.5	215.4	2818.4	679.2	1691.0
	GSA 19 - IS	1095.0	25.7	328.5	0.0	0.0	0.0	0.0	0.0	0.0	0.0	0.0	0.0	1095.0	263.9	657.0
	**Total**	**7372.5**	**173.3**	**2211.7**	**6988.1**	**164.2**	**2096.4**	**5074.5**	**1223.0**	**3044.7**	**4396.8**	**1059.6**	**2638.1**	**23831.9**	**5743.5**	**14299.2**
**Trawl nets**	GSA 09 - NT	294.2	2.8	88.2	259.5	2.5	77.9	228.0	35.0	45.6	193.0	29.6	38.6	974.7	149.5	194.9
	GSA 10 - ST	0.0	0.0	0.0	0.0	0.0	0.0	0.0	0.0	0.0	592.0	90.8	118.4	592.0	90.8	118.4
	GSA 11 - SR	0.0	0.0	0.0	102.0	1.0	30.6	101.0	15.5	20.2	0.0	0.0	0.0	203.0	31.1	40.6
	GSA 16 - SC	576.0	5.5	172.8	915.0	8.7	274.5	466.0	71.5	93.2	882.0	135.3	176.4	2839.0	435.4	567.8
	GSA 17 - NA	2256.1	21.4	676.8	1597.9	15.2	479.4	2194.0	336.5	438.8	2555.8	392.0	511.2	8603.8	1319.6	1720.8
	GSA 18 - SA	1523.6	14.5	457.1	1892.7	18.0	567.8	1618.1	248.2	323.6	1067.6	163.7	213.5	6102.1	935.9	1220.4
	GSA 19 - IS	0.0	0.0	0.0	241.0	2.3	72.3	0.0	0.0	0.0	540.0	82.8	108.0	781.0	119.8	156.2
	**Total**	**4649.8**	**44.2**	**1394.9**	**5008.2**	**47.6**	**1502.5**	**4607.2**	**706.6**	**921.4**	**5830.4**	**894.2**	**1166.1**	**20095.6**	**3082.2**	**4019.1**
**Total**	GSA 09 - NT	1754.5	37.1	526.4	2359.4	155.9	707.8	2251.8	397.5	892.4	1096.0	243.9	570.5	7461.7	1491.9	3438.9
	GSA 10 - ST	2369.0	75.8	710.7	2033.6	47.8	610.1	1256.7	292.6	724.0	2669.0	591.3	1364.6	8328.3	1927.1	4677.7
	GSA 11 - SR	0.0	0.0	0.0	1538.2	42.9	461.5	101.0	15.5	20.2	0.0	0.0	0.0	1639.2	370.0	881.0
	GSA 16 - SC	1159.0	31.6	347.7	1875.0	141.9	562.5	586.1	88.1	129.2	882.0	135.3	176.4	4502.1	714.8	1209.2
	GSA 17 - NA	3987.1	62.1	1196.1	3620.0	83.7	1086.0	3822.8	729.1	1416.1	3646.6	654.9	1165.7	15024.7	2861.0	5549.9
	GSA 18 - SA	1948.6	25.4	584.6	2533.1	43.2	759.9	3108.1	607.3	1217.6	1426.6	250.3	428.9	9016.5	1628.5	2940.3
	GSA 19 - IS	1638.9	101.2	491.7	1155.0	129.1	346.5	2532.4	351.4	759.7	1041.3	152.4	258.4	6367.5	1006.9	2160.7
	**Total**	**12857.2**	**333.3**	**3857.2**	**15114.4**	**644.4**	**4534.3**	**13658.9**	**2481.4**	**5159.3**	**10761.5**	**2028.0**	**3964.5**	**52340.0**	**10000.1**	**20857.6**

**Notes.**

NT North Tyrrhenian ST South Tyrrhenian SR Sardinia SC Sicily Channel NA North Adriatic SA South Adriatic IS Ionian Sea

The mortality rates obtained from fishermen’s interviews enabled to estimate a total of about 10,000 turtle deaths, most of them due to set nets (5,743) and trawl nets (3,082). By applying the mortality rates reported by Casale (2011) it is possible to estimate that about 21,000 turtles can die every year mainly due to set nets (around 14,000 deaths) and trawl nets (around 4,000 deaths).

**Figure 2:**
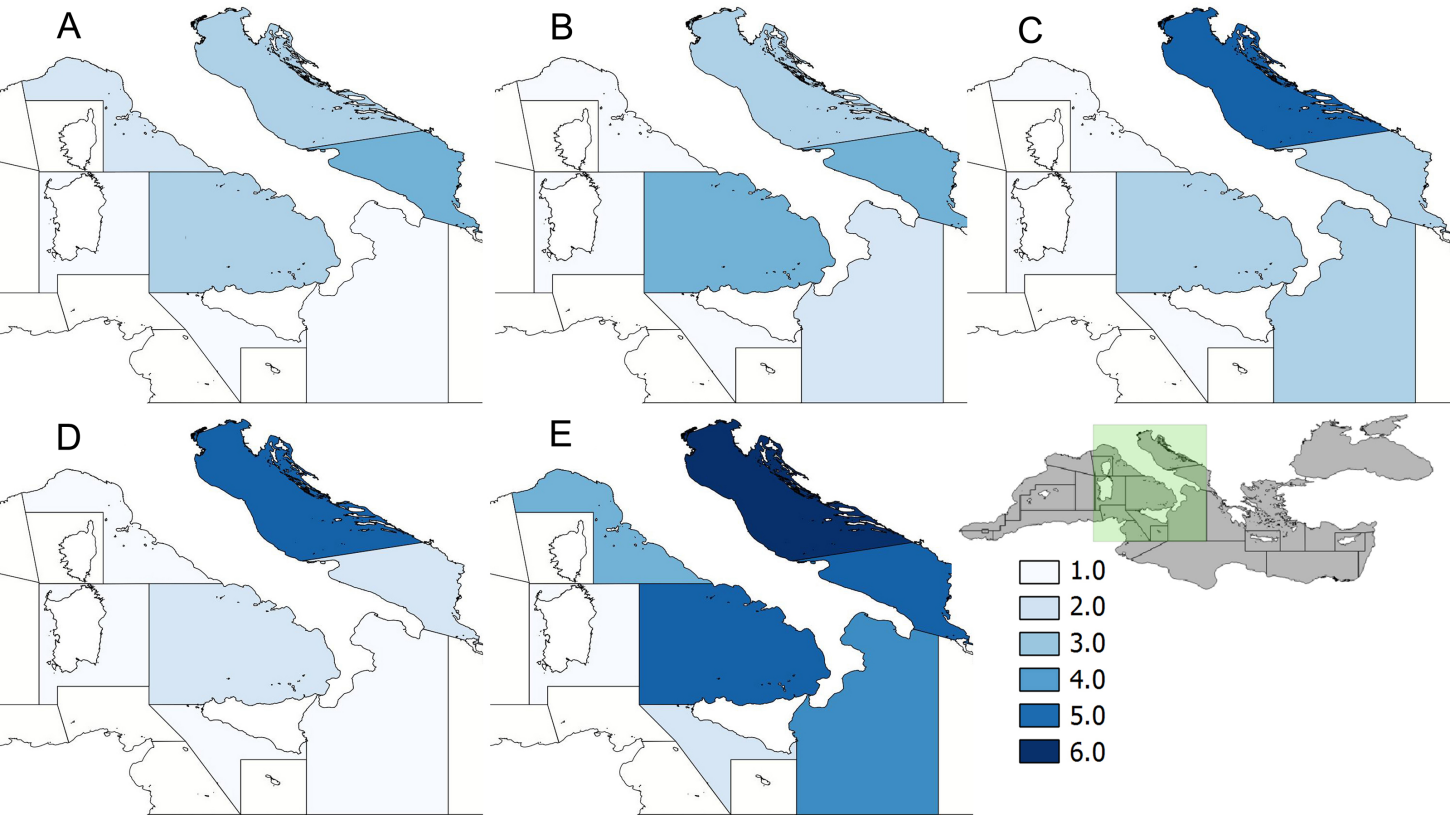
Turtle strandings. Data on stranded turtles ranked using a 6-point scale from 1 (low stranding: 0–25 turtles) to 6 (high strandings: >150 turtles). Source: “Reparto Ambientale Marino”, Italian Ministry of the Environment. (A) Winter; (B) Spring; (C) Summer; (D) Autumn; (E) Total seasons.

The data on stranded turtles, which were especially high in summer and autumn (Fig. 2), confirmed that the Adriatic Sea is the area mostly affected by incidental catch.

**Figure 3:**
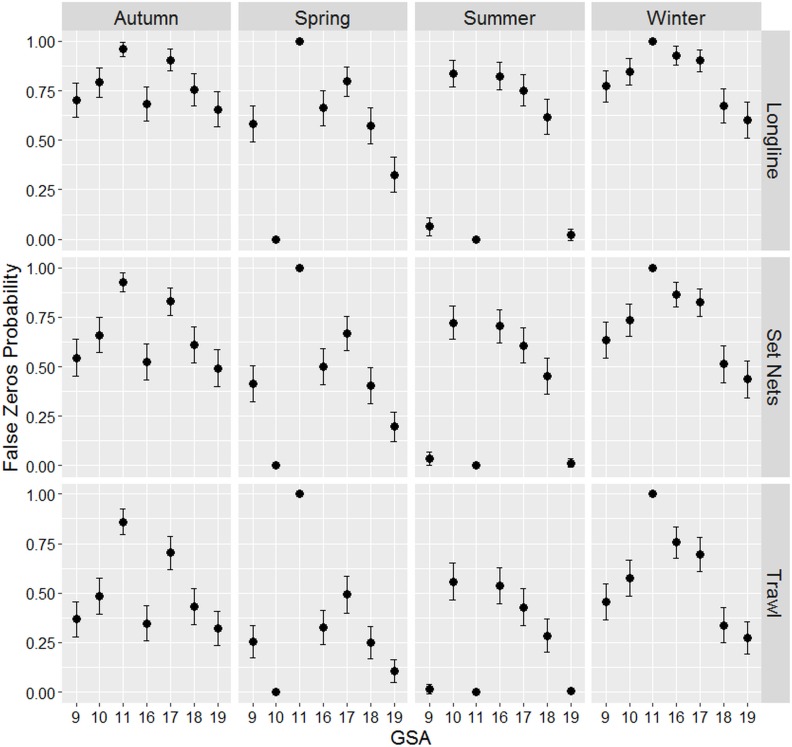
Probability of false zeros. Estimation of the probability of false zeros in the data set measured by the Zero Inflated Negative Binomial (ZINB) model. Bars represent standard errors.

The results from ZINB model are summarized in Table 4. All the factors were highly significant. The factor Season did not appear in the count part of the model indicating that it did not influence on the amount of turtle bycatch. On the other hand, Season appears as an important factor, together with the GSA and Gear, in modelling the zero distributions determining the probabilities to find false zeros. In Fig. 3, the probabilities to observe false zeros (the “zero” part of the model) are shown. The longline showed the highest probabilities that the zeros counted were false zeros in all seasons with the exceptions of summer for GSAs 9, 11 and 19. Regarding the passive nets, the probabilities of false zeros are lower than the previous although still high with only few GSAs showing less than 50% of probabilities, especially in summer and spring. On the contrary, trawl nets showed the lowest probabilities (less than 50%) to record false zeros in all seasons.

**Table 4:** Factors used in the ZINB model. Factors used in the ZINB model and their significance. Count part refers to the GLM negative binomial part of the model for count predictions, while zero part refers to the GLM binomial to predict the probabilities of false zeros.

Factors	Chi^2^	*df*	*p*
**Count part**			
Gear	198.75	32	<0.0001
GSA	155.91	36	<0.0001
GSA*Gear	62.39	12	<0.0001
**Zero part**			
GSA	131.92	24	<0.0001
Season	184.97	21	<0.0001
Gear	18.9	2	<0.0001
GSA*Season	55.52	18	<0.0001

**Figure 4:**
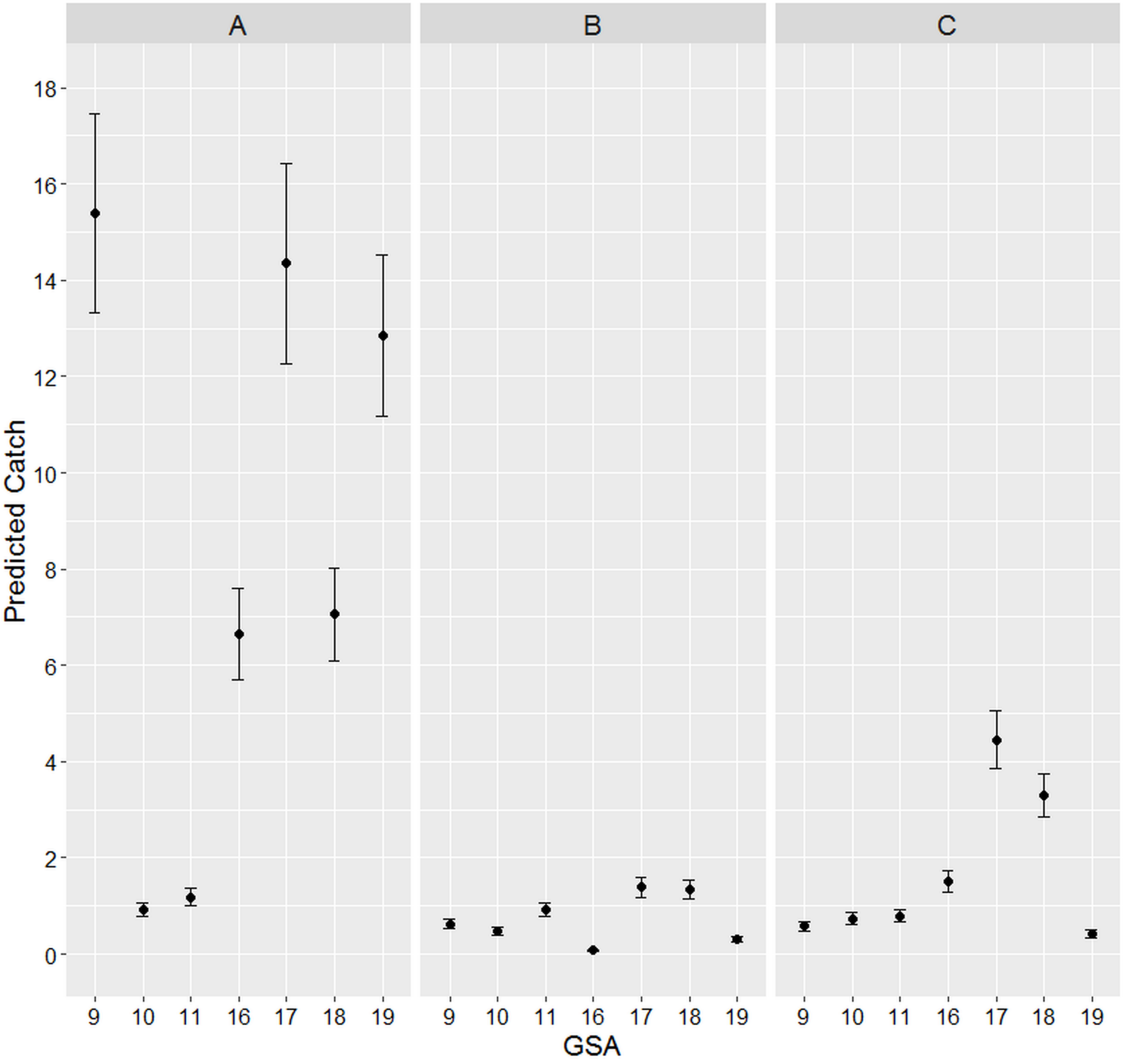
Estimated count. ZINB estimated turtle bycatch per vessel count prediction values for the three gears for each GSA. Bars represent standard errors of the predicted values. (A) Longlines; (B) Set Nets; (C) Trawl Nets.

Figure 4 shows the catch predicted values per boat of the count part of the ZINB model. The highest predicted values were observed for the longline fishery especially in GSAs 9, 17 and 19. Smaller values were observed for the other two gears showing similar catching values between them, although in GSAs 17 and 18 trawl net estimates were higher.

**Figure 5:**
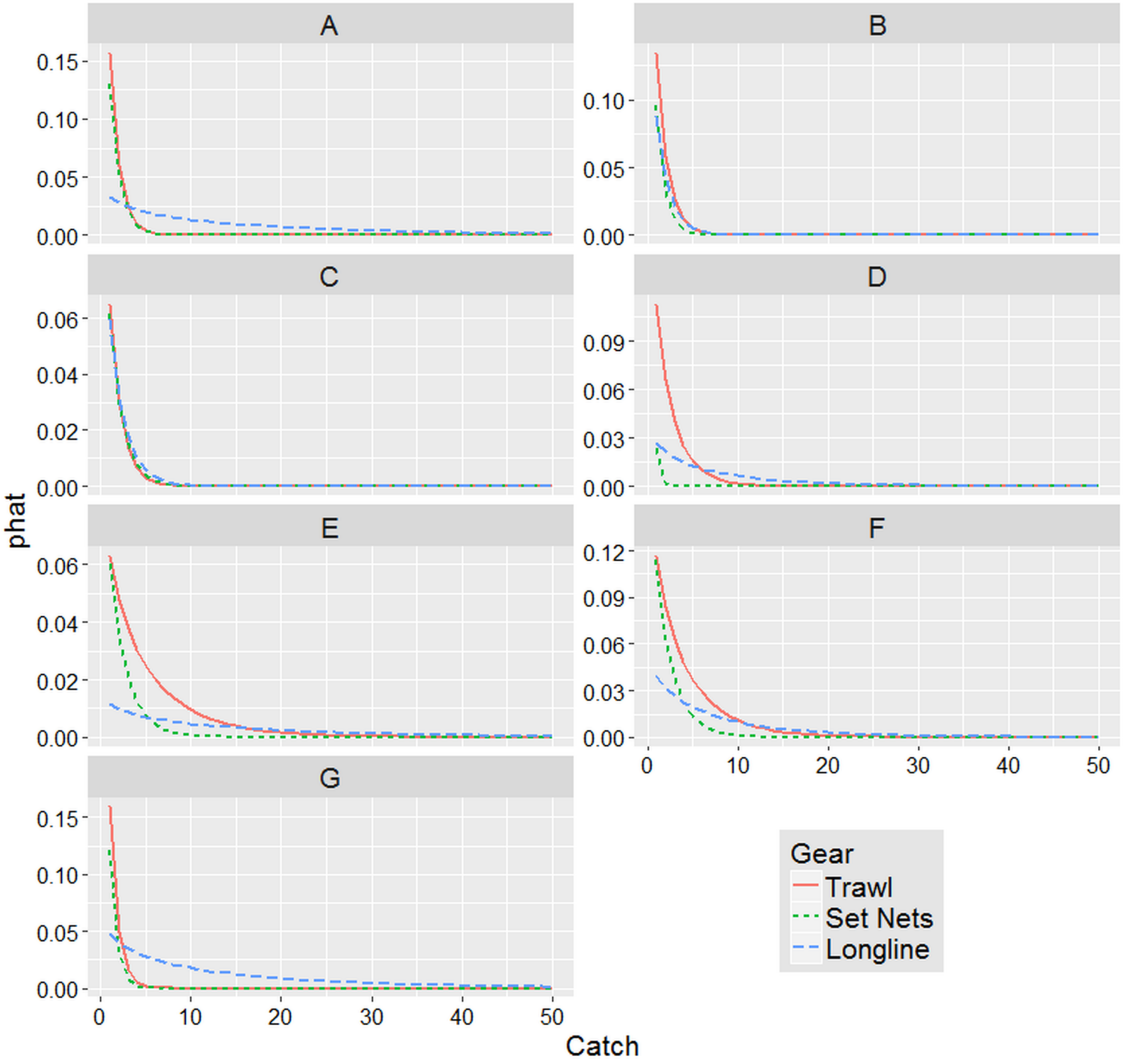
Model estimated probabilities. ZINB model estimated probabilities (phat) of turtle bycatch events in the GSAs. The graphs are zero truncated to highlight the probabilities associated with a positive event. (A) GSA 9; (B) GSA 10; (C) GSA 11; (D) GSA 16; (E) GSA 17; (F) GSA 18; (G) GSA 19.

Figure 5 reports the probabilities to catch turtles calculated by the count part of the ZINB model. What emerged was that, although, the longline predicted values were the highest, the probabilities to catch turtles by means of this gear are very low in all the GSAs (almost < 5%, except in the GSA 10). In other words, it seems that longlines have less probability to bycatch turtles but when they do that it is in relatively massive amounts. On the contrary, notwithstanding the low predicted values for the other two gears, their probabilities to accidentally catch turtles are extremely higher than longlines (between 6 and >15% for trawl nets and between 3 and 12% for passive nets).

Following these results it is clear that the major risks of turtles bycatch were associated to the trawl nets in all the GSAs (especially in the GSAs 9, 19, 18 and 17) followed by the passive nets (apart GSA 16 where the bycatch probabilities were very low) and lastly the longlines showed very low probabilities to catch turtles except in GSAs 10 and 11 where all the three gears showed almost the same probabilities.

**Figure 6:**
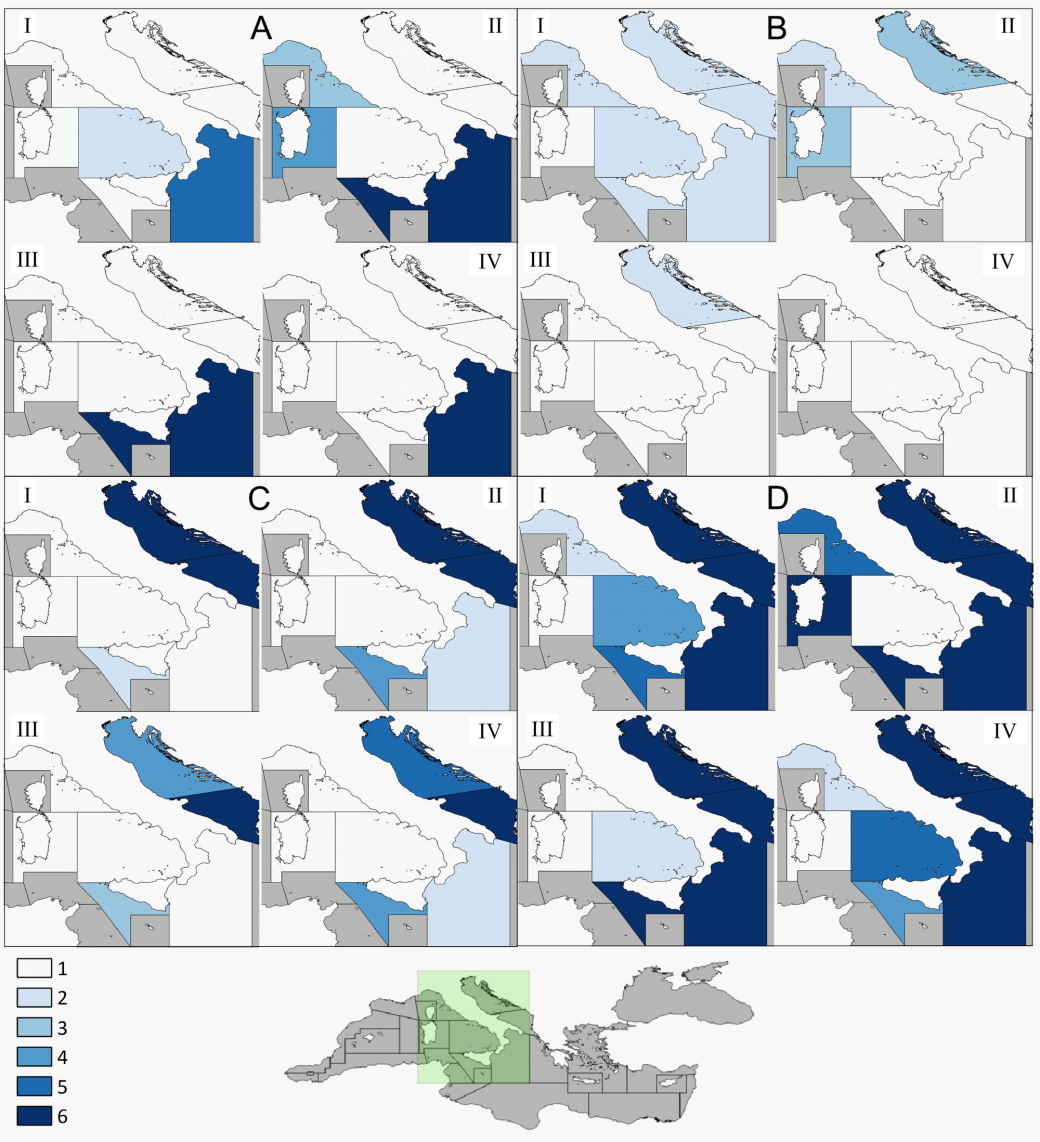
Interaction index. Sea turtle bycatch/gear interaction categorized by gear type and season. Interactions were ranked from 1 (lowest risk of interaction: 0–0.018) to 6 (highest risk of interaction: >0.08). (A) Longlines; (B) Set Nets; (C) Trawl Nets; (D) Total gears. I, Spring; II, Summer; III, Autumn; IV, Winter.

The interaction matrix identified the gears, areas, seasons at the highest risk of bycatch (Fig. 6).

Longlines pose a risk especially in GSA 19 and, to a lesser extent, GSAs 9 and 19 in summer. Interactions with set nets cause the greatest concern in GSAs 17 and 10 in summer. Finally, the whole Adriatic Sea is an interaction hotspot for trawl nets, especially in spring and summer.

### The fishermen’s perspective

#### Turtle encounters and fishermen’s behaviour

The turtle encounter frequency reported by those interviewed is reported in Table 5. Most fishermen (75%) stated that they had caught at least one turtle in 2014; the lowest number was reported by fishermen using set nets (62%) and the highest number by those using longlines (89%) and trawl nets (88%). About 44% reported a disturbance to fishing activities, set nets (61%) and longlines (50%) being more affected than trawlers (26%).

**Table 5:** Interview results in percentages. Interview results in percentages per gear: (1) fishermen that reported at least one capture event and (2) eventual disturbance to fishing activities caused; (3) main causes of disturbance due to turtle catch; (4) fishermen behavior in case of a capture event; (5) fishermen’s opinion and (6) doubts on the adoption of BRDs; (7) fishermen’s awareness of sea turtles conservation issues; (8) fishermen’s level of interest in participation in conservation projects.

ID	Question	Answer	Longlines	Set nets	Trawl nets	Total
1	Catch	YES	82.7	57.6	86.8	71.0
		NOT	17.3	42.4	13.2	29.0
2	Disturb	YES	69.6	60.8	24.3	43.1
		NOT	30.4	39.2	75.7	56.9
3	Cause of disturbance	Reduction of fishing time	46.4	25.5	28.6	28.5
		Damage to fishing gear	14.3	27.0	14.3	21.9
		Damage to fish caught	3.6	13.5	38.8	17.5
		Interruption of fishing activities	17.9	18.4	4.1	17.1
		Others (Bait consuming, etc.)	17.9	15.6	14.3	14.9
4	Behaviour in case of catch	Instantaneous release	47.8	18.9	22.3	24.0
		Delivery to rescue center	39.1	31.1	26.2	29.5
		Release after rest	13.0	50.0	51.5	46.5
5	Opinion on BRD	Yes with money	60.4	33.6	44.1	40.4
		Yes, only training	8.3	19.7	13.6	16.2
		Not, just information	14.6	24.4	16.4	20.3
		Not interested	16.7	22.3	26.0	23.1
6	Doubts and misgiving	Scarse information	36.4	39.9	38.6	39.1
		Fear to change	43.6	37.8	37.1	38.1
		Why change	3.6	9.2	9.6	8.8
		No interst in conservation	16.4	12.7	10.2	12.1
		Other (bureaucracy, regulations, etc.)	0.0	0.4	4.6	1.9
7	Awareness on sea turtle conservation	YES	62.5	63.1	52.3	58.2
		Neutral	33.3	24.5	34.7	29.9
		NOT	4.2	12.4	13.1	11.9
8	Willingness to collaborate in conservation projects	Knowledge of economical benefits	29.9	27.1	35.9	30.3
		Financial programmes for fisheries	22.1	34.7	35.9	33.4
		Make experience with BRDs	26.0	6.1	3.6	7.9
		Knowledge on previous experimental experiences	15.6	8.8	9.4	9.9
		Endangered species protection	2.6	7.9	9.4	7.7
		How to rescue a turtle	3.9	15.5	5.7	10.9

The main disturbances to fishing activities due to incidental turtle catches reported by fishermen were grouped into 5 categories (Table 5). Waste of time was the most common problem reported by fishermen, regardless of fishing method, and was worst in longline fisheries (46%). Turtles were sometimes perceived as competitors and a cause of gear damage, especially during net hauling and disentangling operations (27% of fishermen using set nets). Catch damage and depredation were reported by fishermen using trawl nets (39%) and set nets (27%). In longline fisheries, bait consumption was a cause of concern to 18% of those interviewed. Another cause of disturbance was the fear of Coast Guard inspections and sanctions. A small number of fishermen denied any disturbance due to turtle bycatch and any concern except for the animal’s health.

Direct mortality seems to be low, since 85% of fishermen stated that turtles are usually released in good health conditions (75–100% are alive). Fishermen reported that the direct mortality seems to be high enough for set nets and to a lesser extent, for longlines, while turtles caught with trawl nets are generally released alive (Table 3).

When asked about on board practices (Table 5), 24% of fishermen said that turtles are released immediately, 30% that they are handed over to the Coast Guard or Rescue Centres, and 46% that they are released after allowing them to rest for a short time. Most fishermen reported they were worried about handing the turtles over to the Coast Guard due the bureaucratic hassles and the time wasted, apart from the possibility of catch and vessel inspections. Whereas. the fishermen using set and trawl nets reported that they generally release turtles after a short rest (about 2 h), the longliners stated that turtles are usually released immediately or delivered to Rescue Centres.

The questionnaire showed that fishermen had no clear perception of the annual trend of sea turtle abundance, since 40% denied noting any difference over the past few years, whereas 26% and 33% stated that the population is decreasing and increasing, respectively.

#### Suggestions to reduce turtle bycatch and knowledge of BRDs

Nearly all the interviewees felt that applying mitigation devices (BRDs) to traditional fishing gear would be more effective in reducing turtle bycatch rates than moving to another fishing area. When asked about the possibility of BRD adoption in the Mediterranean, half of the fishermen were in favour of it, albeit only under certain conditions (Table 5), and 40% of these, especially the longliners, believed that incentives would be needed. Of those who were not in favour, only 23% failed to qualify their reply. When fishermen were asked about their chief doubts and misgivings, regarding BRD adoption, two main stances emerged (Table 5), as 39% stated that the main problem was a lack of information and BRD knowledge and 38% were worried about modifying their traditional gear, especially for the fear of performance loss. Another important concern was that BRDs or other similar solutions might become mandatory in the future.

#### Fishermen’s awareness and attitude regarding turtle conservation

Fifty eight percent of the interviewees were aware that their actions could adversely affect sea turtle populations, and that something can be done to preserve the species (Table 5). However, many (30%) were sceptical about the fishermen’s ability to change things. Only 12% replied that fishermen’s behaviour and practices and fishing activities do not affect turtle survival.

#### Participation and cooperation

Many of those interviewed said they would be interested in participating in conservation research projects only in presence of economical rewards: funding programmes (30%) and economical benefits linked to the adoption of sustainable fishing systems (33%; Table 5). A small fraction were interested in technical information, such as BRD use (8%) and the experimental test results obtained with BRDs (10%). The latter proportions were largely similar among fishermen using trawling and set nets (4 and 6% respectively), whereas those using longlines were more interested in gaining knowledge about BRDs from those who have already tried them (e.g., circle hooks) and in learning the experimental test results obtained with BRDs. In general, there seems to be a lack of interest in participating in projects whose main purpose is the protection of endangered species and only some fishermen seemed genuinely interested in learning about the rescue procedures to save the turtles.

## Discussion

This study was devised to collect data on sea turtle bycatch, the threat posed by fishing gears, seasonal and spatial bycatch hotspots in the central Mediterranean Sea (Italian waters) using face to face interviews with fishermen. Turtles-fisheries interactions occur wherever fishing activities overlap with turtle habitats (Lucchetti et al., 2016). Different gears seem to involve different capture and mortality rates (Gerosa & Casale, 1999) and to affect different life stages. The interview data leave no doubt on the scale of the turtle bycatch in most Italian GSAs, where hotspots can also be identified in relation to season and gear type, and where the scenario that is thus outline, is more alarming than earlier studies had led to expect (Casale, Laurent & De Metrio, 2004; Lucchetti & Sala, 2010; Casale, 2011). The present data suggest that more than 52,000 capture events and 10,000 deaths could occur in 2014 in Italian waters alone. An even worse scenario can be obtained if the mortality rates reported by Casale (2011) are applied to our figures (more than 20,000 turtles may be killed incidentally in Italy each year).

Among the fishing gears, trawl nets appear to be the most dangerous in terms of turtle bycatch, with the highest probabilities of bycatch events in all the GSAs. Turtle bycatch estimates highlight a situation of great concern particularly for the GSAs 17 and 18. Also passive nets seem to pose a threat for the conservation of sea turtle in the Mediterranean; the probabilities of bycatch in most of GSAs is high, although estimated catch amount per boat seems to be very low. On the other hand, longlines seem to be the most massive catching gear (catch per vessel, particularly in some areas) but the probabilities of positive events are extremely low and generally lower than the other two gears.

Intense trawl net-loggerhead turtle interactions have already been described in the Northern Adriatic in a study combining fishing effort data and satellite data from tagged turtles (Lucchetti et al., 2016). The area is characterized by shallow waters (<100 m) and rich benthic communities, and is considered as a key-feeding habitat in the whole Mediterranean, where turtles in the demersal stage spend the winter (Lazar & Tvrtkovic, 1995; Casale, Laurent & De Metrio, 2004; Lazar, Ziza & Tvrtkovic, 2006; Lucchetti & Sala, 2010). However, the Northern and Central Adriatic are also characterized by a wide continental shelf, low depth, and a flat seabed, which is ideal for trawling (Lucchetti, Punzo & Virgili, 2016). The high density of turtles and trawlers in autumn and winter in this area give rise to a bycatch hotspot, also supported by the ZINB analysis that highlighted how trawl net was perhaps the most dangerous gear in terms of predicted catches per boat.

The Ionian Sea is a bycatch hotspot in spring and late summer-autumn due to longline fisheries, particularly drifting longlines targeting swordfish (*Xiphias gladius*) and albacore (*Thunnus alalunga*), as reflected by the fishing effort data and supported by the interaction matrix and ZINB predicted values. Loggerhead turtles spend their pelagic stage in this area, feeding on pelagic prey, and cross it on their way to and from the Eastern Mediterranean basin (Lucchetti & Sala, 2010). Other studies have reported that drifting longlines deployed over the continental shelf and in offshore waters are among the main threats to sea turtles in the Mediterranean (Gerosa & Casale, 1999; Margaritoulis et al., 2003; Deflorio et al., 2005). Notwithstanding, the probabilities of catching turtles in GSA 19 were higher for passive and above all for trawl nets, according to the present results.

The turtle bycatch of set nets is usually difficult to assess, because they are operated by a large number of small boats disseminated along the whole Mediterranean coastline. For these reasons, the literature available for these fisheries is scarce. Set nets seem to pose a moderate threat in summer in the Northern and Southern Adriatic and in Sardinia, showing the highest predicted values of catches, although the highest probabilities to encounter a turtle were for GSA 18. The present study confirmed thus the concern expressed by other researchers (Lazar, Ziza & Tvrtkovic, 2006), who estimated that incidental loggerhead bycatch by gillnets in Slovenian and Croatian waters may be as high as 4,038 a year. They also found that gillnets and trammel nets are responsible for high rates of direct mortality, because turtles become entangled when trying to feed on trapped fish and drown because they cannot swim up for air. The Northern Adriatic Sea is thus a bycatch hotspot also due to set nets in summer. A similar concern was expressed by other authors (Casale et al., 2005) who considered the overall interaction between sea turtles and the static net fishery as important as the interaction with the trawl fishery.

Turtle bycatch data collected by direct interviews have the potential to help develop effective conservation measures in the Mediterranean Sea based on the joint effort of fishermen, authorities, and research bodies, as required by recent policies such as the reformed Common Fisheries Policy (EU, 2011) and the Marine Strategy Framework Directive (EU, 2008b).

However, the present results might underestimate the real figure of sea turtle bycatch in Italy. The main reason is that the bycatch, both of commercial species and of protected species, is usually under-reported by fishermen, presumably because of the perceived negative consequences of accurate reporting. The typical fishermen’s reaction to interviews is that nothing good comes from frankness. The fishermen’s main concern is that reporting high bycatch figures might lead administrators to impose additional restrictions, such as closed seasons or areas. Moreover, fishermen feel that they have gained nothing from supporting earlier similar studies, and that society’s general attitude to fishermen is negative. As a result, interviewees often report minimum bycatch events. This has recently been stressed, among others, by Dmitrieva et al. (2013), who assessed the bycatch of Caspian ringed seals and concluded that yearly bycatch estimates were probably several times or even an order of magnitude smaller than the real figure. For this reason, the bycatch data reported in this paper should be considered as estimates of the real figure that could help to identify areas and periods of high risk of turtle bycatch. The interviews elicited a variety of views on sea turtle conservation as well as the adoption of mitigation devices to reduce the turtle bycatch. Most fishermen are aware that their actions may adversely affect the turtle population and that something can be done to preserve this species. Interviews confirm they are aware that the survival chances of injured turtles can be enhanced by taking them to the harbour, but fear of Coast Guard sanctions and waste of time due to administrative issues are the major concerns.

Many feel that applying BRDs to traditional fishing gears would be more effective in reducing the incidental catch of turtles than changing fishing area or period. However, since they fear that BRDs may become mandatory in the future, most of them said that an incentive-based scheme with financial compensation would be essential for their adoption. Finally, there appears to be no clear perception of the ecological importance of safeguarding sea turtles and other protected species, and the principal means to involve fishermen in protection and conservation seem to be economical rewards.

The interview-based approach here adopted provided bycatch estimates even for those fisheries for which information is usually scarce, unavailable, or even subjective. Moreover, the findings allowed accurate identification of the periods, areas and gears at greatest risk. This approach can easily be replicated to identify the bycatch hotspots of other sensitive species, such as marine mammals or sharks.

Once hotspots are identified, technical measures such as alternative gears, BRDs, alternative fishing tactics (i.e., avoid using certain gears in certain periods) can be applied more efficiently. In this regard, the Adriatic Sea emerges as a Mediterranean region severely affected by sea turtle bycatch. Here, a flexible Turtle Excluder Device (fTED) has recently been tested with promising results, since it achieved two aims: it prevented contact of turtles with the catch and did not affect gear performance (Lucchetti, Punzo & Virgili, 2016).

According to Gavin, Solomon & Blank (2010), present results confirm that in case of poor data, when resources are limited, involving and questioning fishermen and stakeholders may be an effective data collection method. This method can yield data on bycatch sufficient to estimate minimum annual bycatch rates, to identify high-risk gear/location/season combinations, and to prioritize areas for further research and for the introduction of management measures.

##  Supplemental Information

10.7717/peerj.3151/supp-1 Dataset S1 Interview dataset

## Additional Information and Declarations

### Data Availability

The following information was supplied regarding data availability: The raw data has been supplied as Dataset S1.
